# Impact of antirotational implants on cutout risk in cephalomedullary fixation of intertrochanteric fractures in elderly: A retrospective comparative observational study

**DOI:** 10.1097/MD.0000000000050464

**Published:** 2026-08-28

**Authors:** Ömer Can Ünlü, İbrahim Altun, Muhammed Melez, Kürşat Tuğrul Okur, Firat Ozan

**Affiliations:** a Department of Orthopedics and Traumatology, University of Health Sciences, Kayseri City Hospital SUAM, Kayseri, Türkiye; b Department of Orthopedics and Traumatology, Memorial Health Group Kayseri Hospital, Kayseri, Türkiye; c Department of Orthopedics and Traumatology, Trabzon Kanuni Training and Research Hospital, Trabzon, Türkiye.

**Keywords:** cutout, femur, intramedullary nailing, mechanical complications, proximal femoral fractures

## Abstract

This study compared the impact of 2 antirotational implants on cutout risk and mid-term clinical outcomes in elderly patients with intertrochanteric femur fractures. Overall, 107 patients (mean age, 80.10 years) who underwent intramedullary nailing for intertrochanteric femoral fractures between April 2018 and December 2020 were included in this study. Patients were divided into 2 groups: those treated with Antirotator Proximal Femoral Nail (A-PFN; group A) and those treated with Interclaw Proximal Femoral Nail Antirotation (group I). This study evaluated lag screw positioning using the Bosworth–Cleveland scale, reduction quality based on Baumgartner’s criteria, tip-apex distance (TAD) values, cutoff values for TAD determined by receiver operating characteristic analysis, time from trauma to surgery, complications, and Harris Hip Score at the final follow-up. The mean follow-up durations were 18.4 ± 8.8 and 16.15 ± 4.72 months in groups A and I, respectively; operative times were 89.38 ± 30.45 and 71.15 ± 27.87 minutes, and time from trauma to surgery was 1.94 ± 1.52 and 1.56 ± 1.12 days, respectively. Nine patients required revision surgery because of implant failure: 2 nail breakages, 6 cutouts, and 1 periprosthetic fracture. No significant difference in complications was observed between the groups (*P* = .812). Baumgartner’s criteria showed good results in 86.8% and 89.7% of groups A and I, respectively (*P* = .765). The Cleveland scale indicated good outcomes in 79.4% and 69.2% of patients, respectively (*P* = .237). Mean TAD was 23.6 ± 1.14 for group A and 25.7 ± 1.19 for group I (*P* = .432), with a receiver operating characteristic analysis cutoff of 31.3. Harris Hip Score showed a stronger functional advantage in group I, with 85.5 points versus 77.5 points in group A (*P* < .001). While both implants provided effective fixation, proper screw placement and anatomical reduction remained key factors in minimizing cutout risk, regardless of implant design.

## 1. Introduction

As the global population ages, the incidence of osteoporotic intertrochanteric fractures (ITFs) is increasing, thereby posing a major societal concern.^[1,2]^ In 2010, hip fractures affected an estimated 2.7 million individuals annually, with 75% of cases occurring in women.^[3]^ A 2019 study by the Global Burden of Disease reported an incidence of 681 hip fractures per 100,000 individuals over 55 years of age.^[4]^ With an aging population, the number of hip fractures is estimated to double over the next 20 to 30 years.^[5]^

In the elderly, ITFs are associated with serious complications such as pressure sores, venous thrombosis, and exacerbation of systemic conditions during prolonged bed rest, contributing to an annual mortality rate of approximately 20%.^[5,6]^ Timely surgical intervention and early mobilization are crucial for preventing mortality.^[7,8]^

Additionally, early intervention can help prevent the mechanical complications associated with ITF fixation. In recent years, intramedullary fixation has gained popularity owing to its biomechanical advantages, including reduced bending moments and enhanced force transmission along the femoral neck.^[9,10]^ Various implant designs, such as proximal femoral nails (PFNs), gamma nails, and InterTAN nails, are currently used to treat ITFs, with different types of PFNs chosen based on fracture stability.^[9]^ However, the development of implants with antirotational properties has been a significant advancement in orthopedic surgery. Implant design plays a critical role in preventing complications such as cutout and reduction loss. These implants increase the penetration of the femoral head to prevent complications, such as implant loosening and shearing, in patients over 65 years of age.^[9–16]^

**Figure 1.**
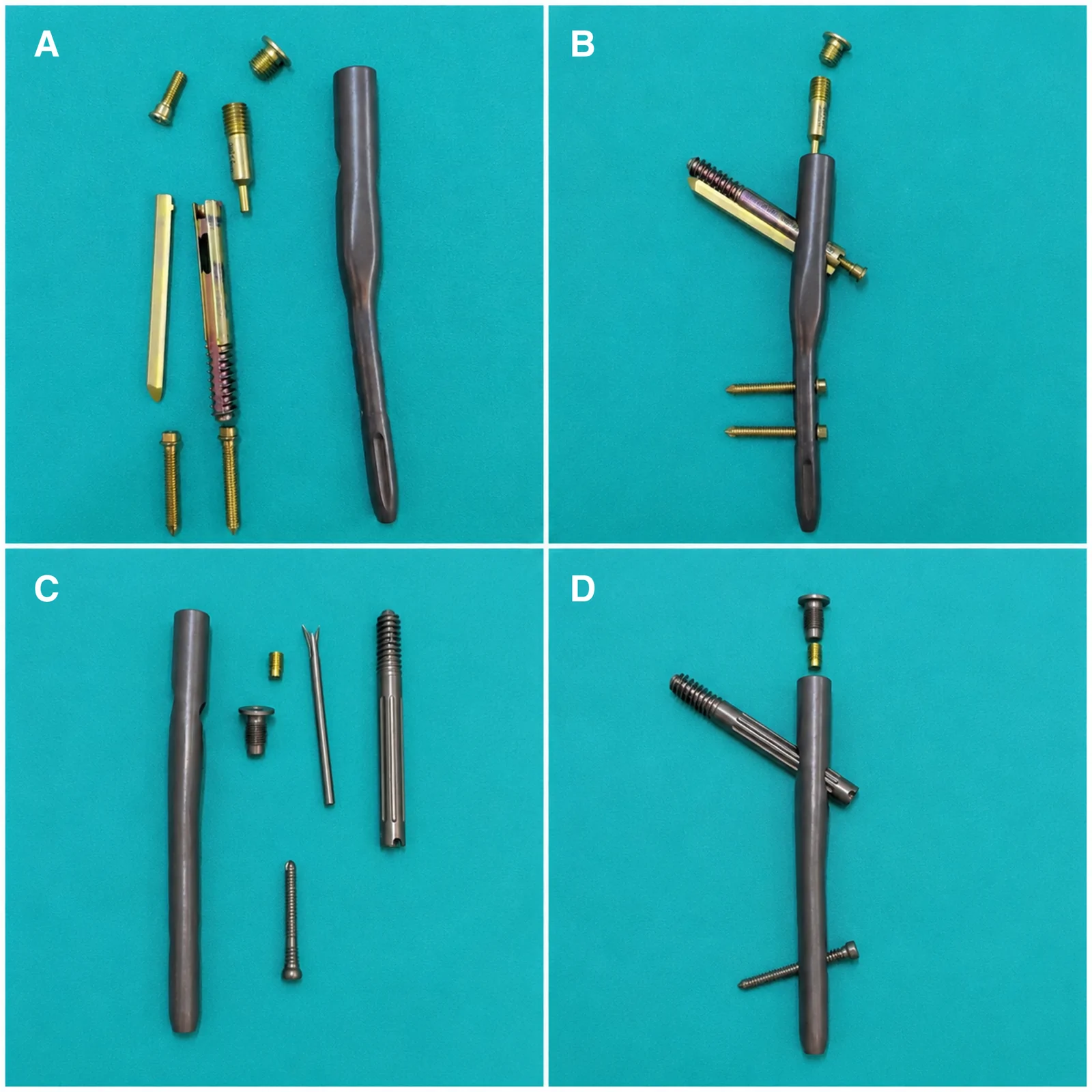
Assembled and disassembled views of the 2 implant systems: (A, B) the first implant and (C, D) the second implant. (A) A separate view of all the components of the Antirotator Proximal Femoral Nail (A-PFN). (B) A-PFN has a 6-degree proximal curvature, a proximal diameter of 16 mm, and a collodiaphyseal angle of 125 degrees, allowing up to 7 mm of compression. (C) A separate view of all the components of the Interclaw Proximal Femoral Nail Antirotation (IPFNA) apparatus. (D) IPFNA is a titanium alloy and has a 6-degree proximal curvature, a proximal diameter of 16.5 mm, and a 130-degree collodiaphyseal angle.

This study compared the Interclaw Proximal Femoral Nail Antirotation (IPFNA; Zimed, Gaziantep, Turkey) and Antirotator Proximal Femoral Nail (A-PFN; TST, Pendik, Istanbul, Turkey) devices (Fig. 1). Both are intramedullary implants that provide rotational stability to osteoporotic bone. The A-PFN uses a lag screw with rotational control, whereas the IPFNA uses a claw-like design with a neck-shaft angle of 130°. Few studies have compared the radiological and functional outcomes of these implants in elderly patients.

This study aimed to compare the clinical and radiological outcomes of 2 intramedullary fixation devices (A-PFN and IPFNA) for intertrochanteric femoral fractures. As a secondary aim, we investigated the effects of quality of reduction on postoperative complications.

## 2. Materials and methods

### 2.1. General data

**Figure 2.**
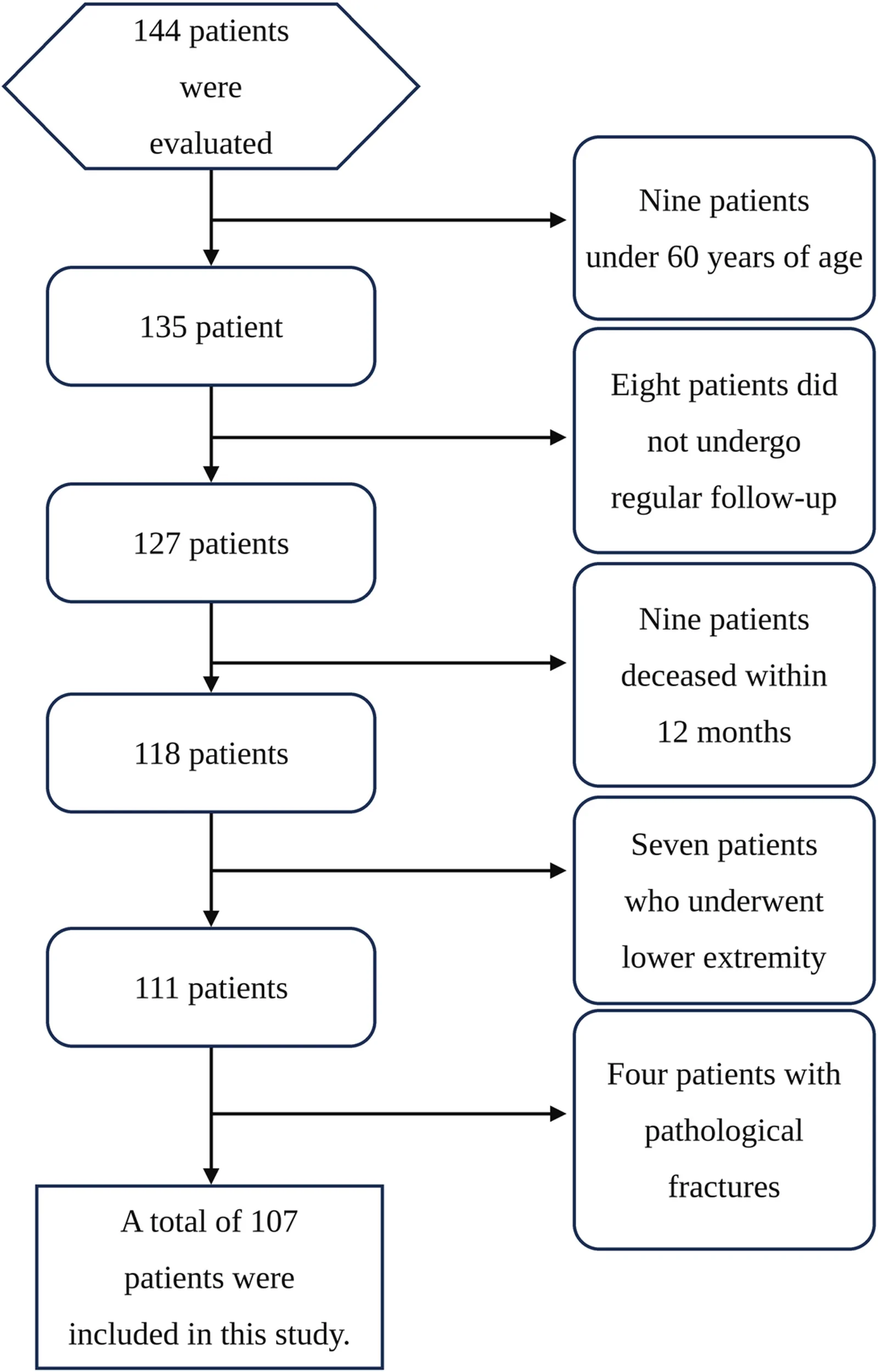
Evaluation of patient numbers after applying exclusion criteria.

**Figure 3.**
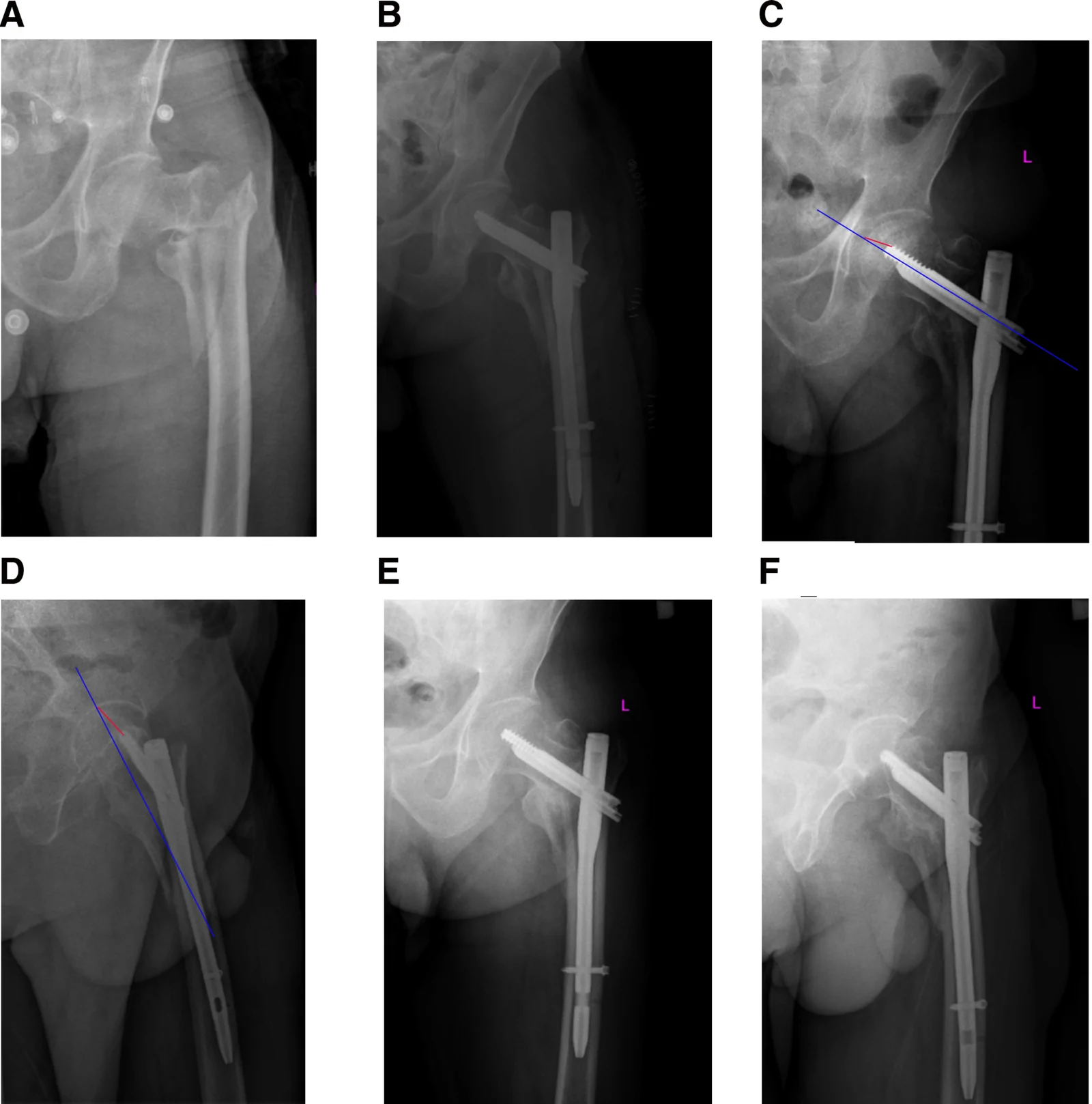
Radiographic follow-up of a patient treated with an A-PFN implant: a 67-year-old male with fragmented intertrochanteric fractures (ITFs) and tip-apex distance (TAD) = 33.5 mm. (A) Preoperative radiography. (B) First postoperative day. (C, D) Postoperative third month. (E, F) Postoperative 12th month. Blue line: femoral head type with femoral neck reference. Red line: tip-apex distance. A-PFN = Antirotator Proximal Femoral Nail.

**Figure 4.**
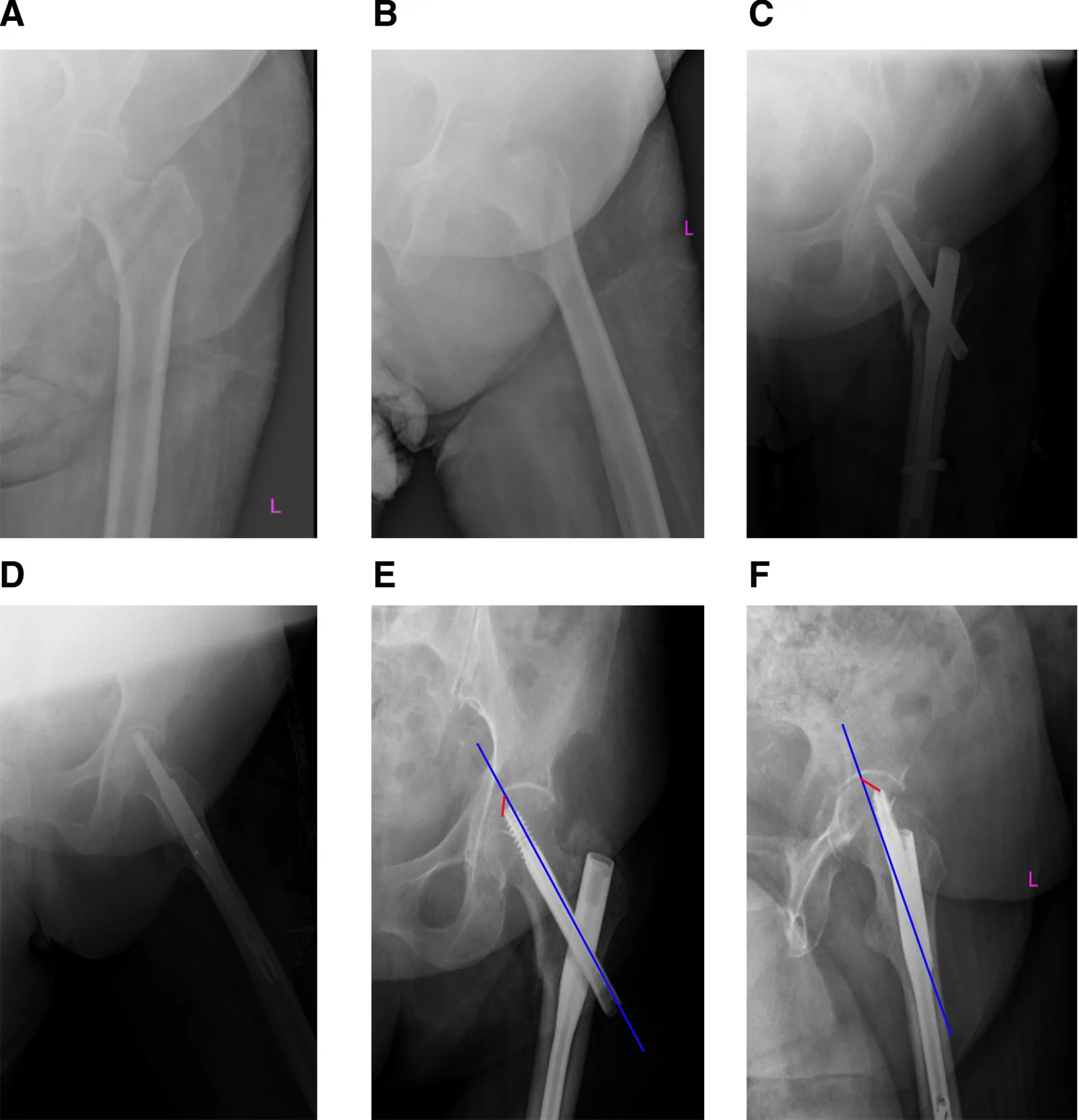
Radiographic follow-up of a patient treated with an IPFNA implant: a 79-year-old male with fragmented intertrochanteric fractures (ITFs), TAD = 29.2 mm, and poor positioning (Cleveland index no. 3). (A, B) Preoperative radiography. (C, D) Postoperative day 5. (E, F) Postoperative 3.5 months. Blue line: femoral head type with femoral neck reference; Red line: tip-apex distance. IPFNA = Interclaw Proximal Femoral Nail Antirotation.

This study included 107 patients aged 60 years or older who underwent closed reduction and intramedullary nailing for intertrochanteric femoral fractures performed by traumatology specialists at the Orthopaedics and Traumatology Clinic of the Kayseri City Training and Research Hospital between April 2018 and December 2020. In our study, the operations were performed by a total of 17 different orthopedic surgeons, who were senior surgeons with at least 5 years of trauma surgery experience, following a standardized institutional protocol. These patients underwent either A-PFN or IPFNA at our institution; only the first cohort of 144 patients with at least 12 months of clinical follow-up data was included in the study. Implant selection was determined by the institution’s purchasing criteria and was chosen by the operating surgeon, with an eye toward antirotation options. Some patients were excluded from the study because factors such as pathological fracture, previous surgery in the lower extremities, lack of regular follow-up, and early death could have led to inaccurate results and misdirection. The inclusion and exclusion criteria are summarized in Table 1, and the excluded patients are depicted in a flowchart detailing the reasons for their exclusion (Fig. 2). Patients were divided into 2 groups based on the type of implant used: group A received A-PFN (Fig. 3), and group I received IPFNA (Fig. 4). The lag screw diameters were 10 to 11 mm for the A-PFN implant and 11 mm for the IPFNA implant, which were considered in the radiographic calibration during tip-apex distance (TAD) measurement. Distal locking was achieved with 2 screws in the A-PFN and 1 screw in the IPFNA, and nail diameters (11–12 mm) were selected according to the femoral canal size.

**Table 1:** Inclusion and exclusion criteria for the study.

Inclusion criteria	Exclusion criteria
Acute fracture (within 5 d after the fracture)	Who died within the first 12 mo following surgery
Over 60 yr old	With irregular follow-ups
With regular radiological follow-up for at least 6 wk and 3 and 6 mo	Who underwent simultaneous fractures of the lower extremities operations
Minimum 12 mo of clinical follow-up	With pathological fractures (osteomyelitis or malignancy)

This study is based on a medical specialization thesis entitled “Comparison of the Results of Antirotation Proximal Femur Nailing and Dyna Locked Trochanteric Nailing in Intertrochanteric Femur Fractures,” numbered 335 by the Turkish Council of Higher Education. The study protocol was approved by the Ethics Committee of Kayseri City Hospital (approval date: March 18, 2021; approval number: 335), and written informed consent was obtained from each patient. This study was conducted in accordance with the principles of the Declaration of Helsinki. The study was also conducted in accordance with the Strengthening the Reporting of Observational Studies in Epidemiology guidelines.

### 2.2. Radiological and clinical assessment

A picture archiving and communication system (KEYDATA, Information Technology Systems, Ankara, Türkiye) was retrospectively used to assess fracture reduction and implant positioning. Postoperative assessments of reduction quality, TAD, implant positioning, osteoporosis, and union status were conducted by the authors using radiographs in a blinded manner. Multiple radiological scales were used to provide a comprehensive assessment: TAD to quantify cutout risk, femoral neck-shaft angle, Parker’s ratios (PRs), Cleveland index to evaluate screw positioning, and Baumgartner’s criteria to assess reduction quality. Radiological evaluations included anteroposterior and lateral views to assess the entire femoral nail. Angulation >5° was considered a varus deformity, and separations in the fracture line >5 mm were considered loss of reduction. Because standardized dual-energy X-ray absorptiometry (DXA) was not available for all patients in this retrospective acute-trauma cohort, osteoporosis was graded on standard anteroposterior radiographs using the Singh index and cortical thickness index. These radiographic indices are inexpensive, reproducible surrogates that correlate with femoral bone mineral density and are widely used when DXA is impractical.^[17–19]^

**Figure 5.**
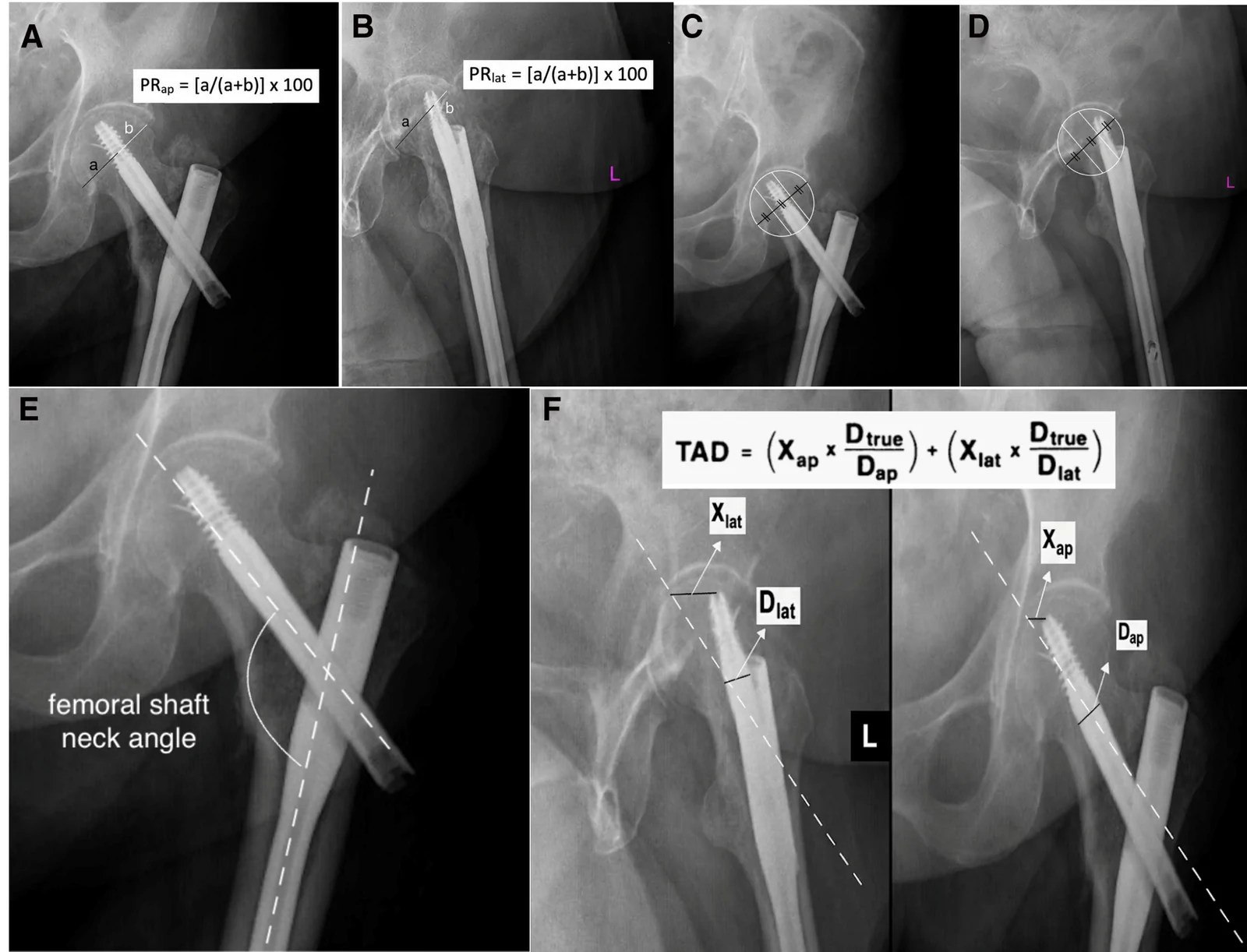
Radiological evaluation methods for implant placement and fracture position after surgery in intertrochanteric femoral fractures. (A) Appearance of measurement and calculation of PR in anterior-posterior radiographs. (B) Appearance of measurement and calculation of PR in a lateral radiograph. (C) View of the Cleveland quadrant on an AP radiograph. (D) View of the Cleveland quadrant on a lateral radiograph. (E) The appearance of the calculation of the femoral shaft neck angle on an AP radiograph. (F) Appearance of measurement and calculation of TAD on radiographs in AP and lateral radiographs. AP = anterior-posterior, D = diameter, Lat = lateral, PR = Parker ratio, TAD = tip-apex distance.

The total TAD, as measured on anteroposterior and lateral radiographs, was identified as the TAD value. Reduction was classified as “good” if both Baumgartner’s criteria were satisfied and “acceptable” if only one condition was satisfied. The Cleveland index, which divides anteroposterior and lateral hip joint radiographs into 9 regions, was used to categorize lag screw positions in the femoral head. According to the Cleveland index, central-central and inferior-central positions were deemed good (5, 6, 8, and 9), whereas other positions were considered poor (1, 2, 3, 4, and 7). According to the PR, favorable implant positioning was achieved if the lag screw was in the lower half of the femoral neck in the anteroposterior view and centrally in the lateral view. Poor positioning was indicated if the screw was in the upper half of the anteroposterior view or at the edge of the lateral view. The radiological measurement and calculation formulas for the Cleveland index, PR, TAD value, and femoral shaft neck angles are shown in Figure 5.

Follow-up assessments, including radiological assessments, were conducted at 6 weeks; 3, 6, and 12 months; and annually thereafter. Fractures were considered healed radiographically if bridging calluses were observed in 3 of 4 cortices in 2 views. Clinical evaluations were performed at 6, 12, and 24 months postoperatively using the Harris Hip Score (HHS). Patient medical records were reviewed to identify functional and biomechanical complications that could affect outcomes, such as operative time, hospitalization duration, venous thromboembolism, pneumonia, urinary tract infection, wound hematoma, surgical site infection, avascular necrosis, revision requirements, and time to union.

### 2.3. Surgical procedure

The patients underwent surgery after the optimal conditions were achieved. After anesthesia was administered, the patients were placed in the lateral decubitus position with the fractured side facing upward. Cefazolin (1 g) was administered intravenously approximately 30 minutes prior to surgery. The fractured lower extremities were sterilized and draped. After reduction was visualized using a C-arm scope, the procedure was performed through the greater trochanter. Initially, lag screws were placed in both nails using a scope and guidewire. The blades of the IPFNA nail were removed after the placement of the lag screw. An underlying antirotation wedge was inserted into the nail. Compression was then applied to both nails, and the lag screw was locked with an apical screw. Each nail was locked distally with a single screw, followed by the insertion of the apical screw. The layers were then properly closed, and the surgery was completed.

### 2.4. Rehabilitation

Hip and knee mobility exercises were initiated on the first postoperative day for all patients under the supervision of a physiotherapist. Patients not hospitalized in the intensive care unit were permitted partial weight-bearing with a walker or crutches on the first day. Full weight-bearing was allowed after the sixth week.

### 2.5. Statistical analysis

All data were analyzed using Statistical Package for the Social Sciences software (IBM Corp., released 2013, IBM SPSS Statistics for Windows, version 22.0, Armonk). A power analysis was conducted to determine the sample size, resulting in a total of 109 participants (tails: 2; effect size: 0.3; α err prob: 0.05; power: 0.90). The power analysis indicated that a minimum of 109 patients were required. The final study cohort included 107 patients, and 2 patients were excluded due to incomplete follow-up. This minor shortfall is unlikely to have affected the statistical power of this study. Categorical data were presented as frequencies and percentages, whereas continuous variables were expressed as mean values with standard deviations. Pearson’s chi-square test was used to compare categorical variables between the groups. The normality of the data distribution was assessed using the Shapiro–Wilk test, skewness and kurtosis values, and histograms. The independent *t* test was used to compare normally distributed continuous variables, with *P*-values below .05 considered statistically significant. Non-normally distributed continuous variables were compared using the Mann–Whitney *U* test. Univariate binary logistic regression analysis was performed to identify factors influencing cutout, including age, sex, implant type, time from admission to surgery, type of anesthesia, surgical time, osteoporosis, TAD, Cleveland index, PR, Baumgartner’s reduction criteria, and fracture type, according to the Arbeitsgemeinschaft für Osteosynthesefragen (AO) classification. Multivariate binary logistic regression analysis was performed using TAD, Cleveland index, PR, and Baumgartner’s reduction criteria, which were found to have a significant effect on cutout in univariate analysis. Receiver operating characteristic (ROC) curve analysis was used to determine the threshold value of TAD because of the similar efficacy of the 2 implants evaluated in the study. As TAD was found to be a statistically significant predictor of cutout in univariate logistic regression analysis, ROC analysis was subsequently performed to determine the optimal cutoff value based on Youden’s J index.

## 3. Results

The patients were followed up for an average of 17.58 ± 7.631 months (range: 6–36 months). Among the patients, 88.8% (n = 95) received spinal anesthesia, 6.6% (n = 18) received general anesthesia, and 4.6% (n = 5) underwent surgery with peripheral nerve blockade. Patients who received a combination of peripheral nerve blockade and spinal anesthesia were categorized as having regional or general anesthesia. In group A, 64 patients had an American Society of Anesthesiologists (ASA) score of 2, and 4 patients had an ASA score of 3. In group I, 32 patients had an ASA score of 2, and 7 had an ASA score of 3. According to the AO criteria, 42% (n = 45) of the fractures were unstable (A2 and A3), and 58% (n = 62) were stable (A1). The mean duration of hospitalization (*P* = .008) and operative time (*P* = .003) differed significantly between groups I and A. When analyzing age, sex, fracture side, time to union, and AO classification between the groups, only the difference in sex was found to be statistically significant (*P* = .034). There was no statistically significant difference between the groups regarding the number of distal locking screws used (*P* = .097) or nail diameters (*P* = .896). Osteoporosis was identified in 53 patients, and the difference between the groups was significant (*P* < .001; Table 2).

**Table 2:** Distribution of patients based on demographic and postoperative clinical data.

Variables	Group A (n = 68)		Group I (n = 39)		*P*
	n	Mean ± SD	n	Mean ± SD	
Age (yr)		81.15 ± 8.32		78.18 ± 8.23	.789*
Sex, female/male	44/24		17/22		**.034** *
Side, right/left	35/33		14/25		.120*
AO classification (%)					
A1	38		24		
A2	16		10		.599†
A3	14		5		
ASA score, II/III	64/4		32/7		.094†
Time from trauma to surgery (d)		1.94 ± 1.52		1.56 ± 1.12	.234*
Osteoporosis	45		8		**.001** †
Operative time (min)		89.38 ± 30.5		71.15 ± 27.9	**.003** *
Hospitalization duration (d)		7.13 ± 4.98		8.15 ± 12.57	**.008** *
Distal locking screw, n		2		1	.097
Nail diameter, mm		10 (10–11)		10 (10–11)	.896
Time to union (wk)		20.28 ± 3.35		21.59 ± 4.28	.147*
Type of anesthesia (%)					
Spinal		60 (88.2)		35 (89.7)	
General		5 (7.4)		2 (5.1)	.91†
Peripheral block		3 (4.4)		2 (5.2)	
Follow-up duration (mo)		18.40 ± 8.81		16.15 ± 4.72	.519*
HHS		74.44 ± 15.2		84.67 ± 5.63	**.001** *
Complication-overall, n (%)	8 (11.8)		4 (10.3)		.812†
Cutout	4		2		.619†
Nail breakage	1		1		1.00†
Periprosthetic fracture	0		1		.364†
Superficial infection	1		0		.475†
Nonunion	2		0		.532†

*P*-values < .05 are shown in bold.

AO = Arbeitsgemeinschaft für Osteosynthesefragen, ASA = American Society of Anesthesiologists, HHS = Harris Hip Score, SD = standard deviation.

* Independent *t* test.

† Fisher’s exact test.

The operative time, defined as the time from anesthesia induction to the patient leaving the operating table (including fracture reduction, patient cleaning, and setup), was significantly longer in group A than in group I, as determined by the independent *t* test (*P* = .003). Fisher’s exact test did not show a statistically significant difference in the type of anesthesia used between the groups. HHS represents the value at the latest follow-up visit, typically between 12 and 24 months postoperatively. Before surgery, all patients were able to mobilize with or without support. However, there was a statistically significant difference in the postoperative HHS scores (*P* < .001; Table 2).

**Figure 6.**
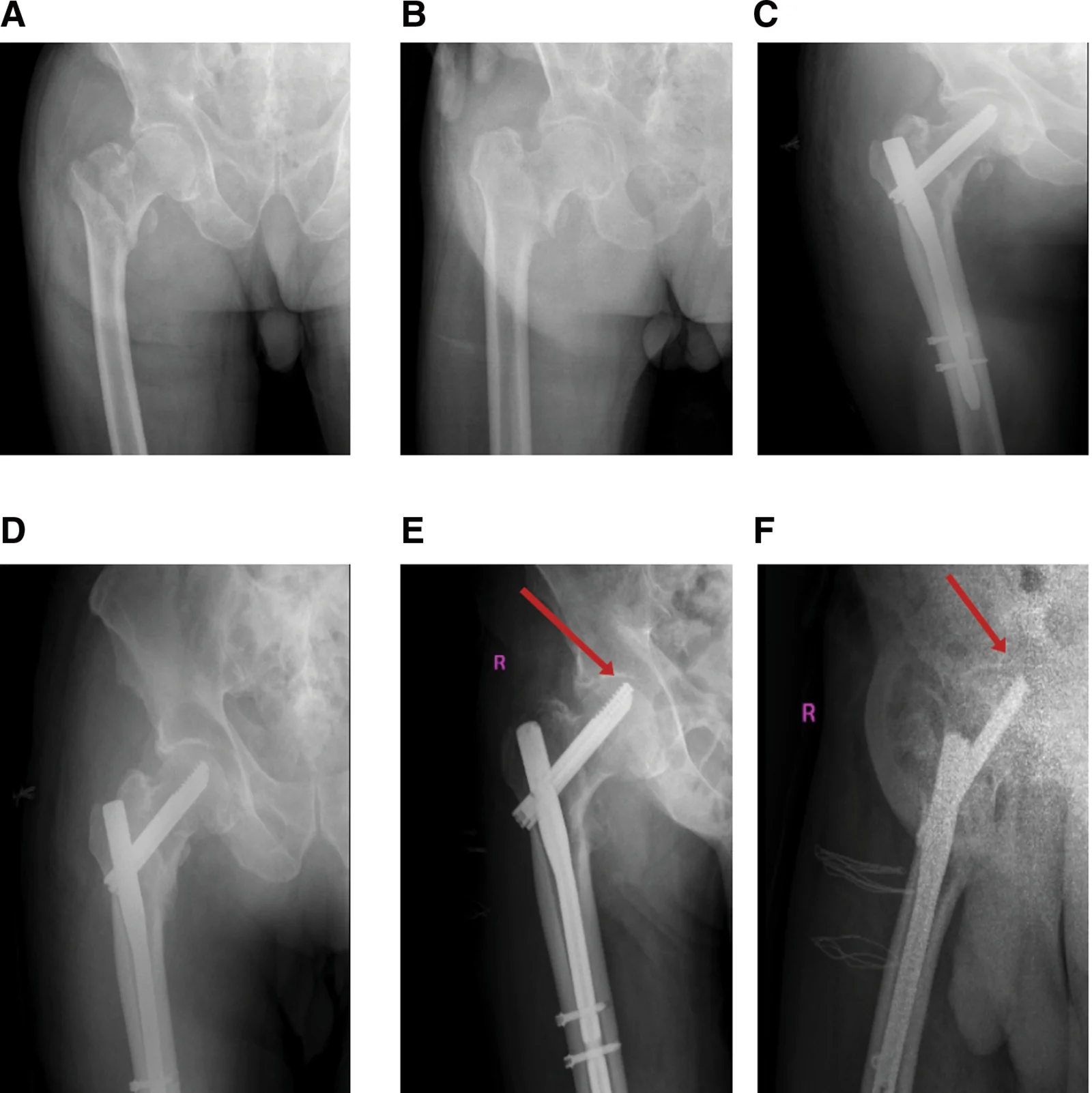
Radiographic follow-up of a patient treated with a cutout IPFNA implant: a 78-year-old male with fragmented intertrochanteric fractures (ITFs), tip-apex distance (TAD) < 25 mm. (A, B) Preoperative radiography. (C, D) First week postoperatively. (E, F) Follow-up at 1 month showing superior cutout of the lag screw through the femoral head. Red arrow indicates that the screw has cut out from the femoral head. IPFNA = Interclaw Proximal Femoral Nail Antirotation.

The overall complication rates were 8 (11.8%) and 4 (10.3%) in groups A and I, respectively (*P* = .812). Among the patients with complications, only 1 patient in group A developed a superficial infection, which was successfully treated with serial debridement, irrigation, and antibiotic therapy. Both groups had 1 case of nail breakage that required revision surgery. In group I, 1 patient experienced a periprosthetic fracture, while in group A, 2 patients with nonunion underwent revision nail surgery. In 5 patients who underwent nail revision, bone healing was achieved. Among patients with unstable fractures, cutout occurred in 4 patients in group A and 2 patients in group I, necessitating hemiarthroplasty surgery (Fig. 6). No cases of avascular necrosis were observed during follow-up. Therefore, comparative statistical analysis for avascular necrosis could not be performed.

**Figure 7.**
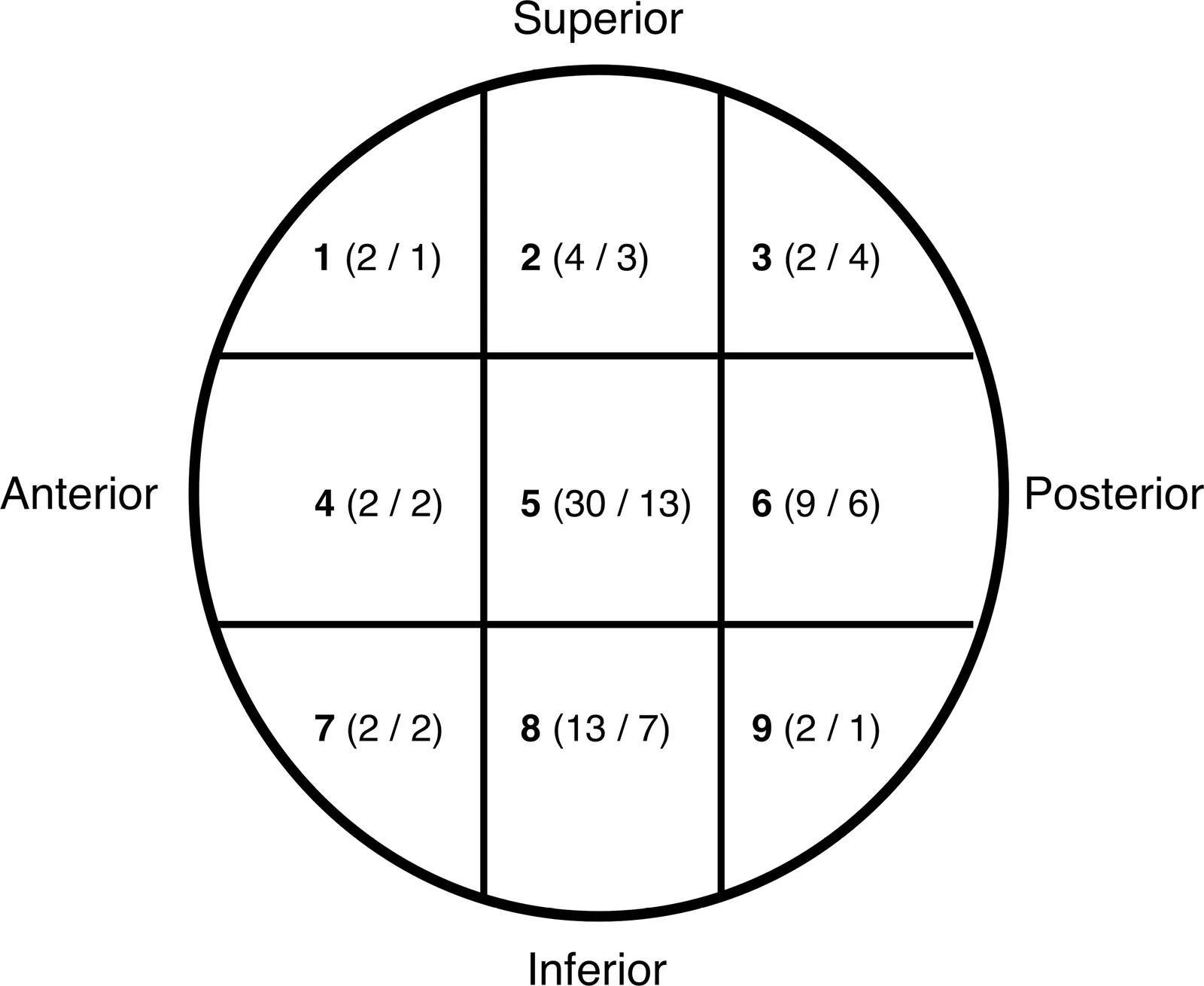
Postoperative radiographic positioning of lag screws, with numbers in parentheses indicating the number of cases for each specific screw placement (group A/group I).

According to Baumgartner’s criteria, good reduction was achieved in 86.8% of group A patients and 89.7% of group I patients, with no statistically significant difference (*P* = .765). Lag screw placement evaluation based on the Cleveland index showed approximately 30% poor positioning (*P* = .237; Fig. 7). The mean TAD values were within the normal limits in each group (normal limit, TAD < 25 mm), with a broad range of values due to outliers; however, the difference between the groups was not statistically significant (*P* = .432). The TAD exceeded 25 mm in 24 cases in group A (cutout in 3 patients, 12.5%) and 15 cases in group I (cutout in 2 patients, 13.3%), with no statistical significance. In one case of cutout, the TAD was <25 mm. The radiological and clinical outcomes of the 6 cases in which cutout occurred are shown in Table 3. The neck-shaft angle showed a significant difference between the groups in the *t* test due to variations in nail angles (*P* = .044), indicating consistency between the femoral neck angle and the nail lag screw angle (Table 4).

**Table 3:** Clinical and radiological results of cutout cases.

Case	Group	Age/sex	AO type	TAD (mm)	Cleveland zone	Parker (%)	Baumgartner/revision
1	A-PFN	79/Male	A3	>25	Poor	68	Not met/hemiarthroplasty
2	A-PFN	76/Female	A2	>25	Poor	47	Not met/hemiarthroplasty
3	A-PFN	83/Female	A2	>25	Poor	70	Not met/hemiarthroplasty
4	A-PFN	74/Male	A2	<25	Good	67	Not met/hemiarthroplasty
5	IPFNA	71/Female	A2	>25	Poor	67	Not met/hemiarthroplasty
6	IPFNA	73/Male	A2	>25	Poor	70	Not met/hemiarthroplasty

Group A = A-PFN; Group I = IPFNA.

A-PFN = Antirotator Proximal Femoral Nail, AO = Arbeitsgemeinschaft für Osteosynthesefragen, IPFNA = Interclaw Proximal Femoral Nail Antirotation, TAD = tip-apex distance.

**Table 4:** Analysis of radiological parameters in 2 distinct patient groups.

Variables	Group A (n = 68)			Group I (n = 39)			*P*
	n	%	Mean ± SD	n	%	Mean ± SD	
Baumgartner’s criteria							.765†
Good reduction	59	86.8		35	89.7		
Poor reduction	9	13.2		4	10.3		
Cleveland index							.237†
Good	54	79.4		27	69.2		
Poor	14	20.6		12	30.8		
Parker’s ratio			50.94 ± 10.6			52.38 ± 11.82	.518*
Femoral neck-shaft angle			129.53 ± 7.24			132.38 ± 6.43	**.044** *
TAD (mm, min–max)			23.6 ± 1.14 (3–70)			25.7 ± 1.19 (3–65)	.432*
TAD							.835†
Below 25 mm	44	64.7		24	61.5		
Above 25 mm	24	35.3		15	38.5		
TAD							.200†
Below 31.3 mm	58	85.3		29	74.4		
Above 31.4 mm	10	14.7		10	25.6		
Varus deformity	2	2.9		2	5.1		.621†
Loss of reduction	2	2.9		1	2.5		1.000†
Loss of femoral angle	4	5.8		3	7.6		.704†

*P*-values < .05 are shown in bold; femoral neck-shaft angle is within the normal range (125 ± 5).

max = maximum, min = minimum, SD = standard deviation, TAD = tip-apex distance.

* Independent *t* test.

† Fisher’s exact test.

Univariate regression analysis identified TAD, Cleveland index, Baumgartner’s reduction criteria, and PR as the parameters affecting cutout (Table 5). Univariate regression analysis for sex (Beginning block variables not in the equation = 0.721, Omnibus tests of coefficients = 0.722, Nagelkerke *R*^2^ = 0.003, *P* = .722) and osteoporosis (Beginning block variables not in the equation = 0.388, Omnibus tests of coefficients = 0.384, Nagelkerke *R*^2^ = 0.020, *P* = .397), which are important parameters in terms of mechanical complications, showed no statistically significant effect and were not included in the multivariate regression model.

**Table 5:** Results of univariate binary logistic regression analyses affecting cutout.

Variables	Regression coefficient	SE	*P*	OR	95% CI
Baumgartner’s criteria	3.018	0.934	**.001**	20.440	3.279–127.47
Cleveland index	1.972	0.899	**.028**	7.182	1.233–41.824
Parker’s ratio	0.121	0.044	**.006**	1.128	1.035–1.230
TAD (mm)*	3.356	1.131	**.003**	28.667	3.126–262.88

*P*-values < .05 are shown in bold.

CI = confidence interval, OR = odds ratio, SE = standard error, TAD = tip-apex distance.

* Risk value when categorized according to a tip-apex distance of 31.3 mm.

**Figure 8.**
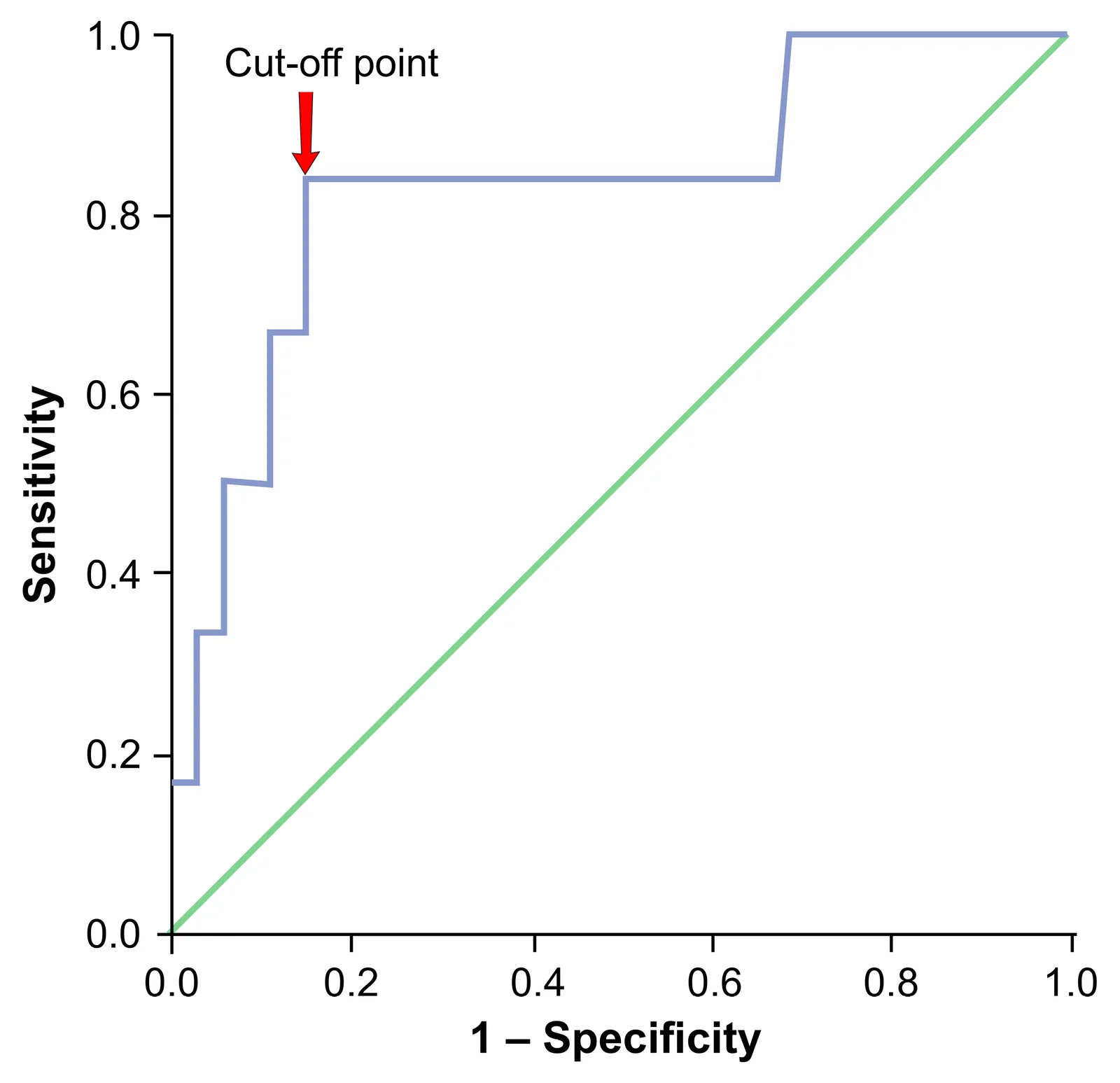
Cutoff graph of tip-apex distance (TAD) values using receiver operating characteristic (ROC) analysis. Red arrow indicates the position of the TAD cutoff value on the graph.

In the multivariate logistic regression analysis, only reduction quality was significant among the 4 parameters associated with the risk of cutout (Table 6). Among the 6 cases (5.6%) with cutout, 5 lag screws were poorly positioned in the Cleveland zones, the TAD values exceeded 25 mm, Baumgartner’s reduction criteria were not fully met, and these cases were exclusively observed in unstable fractures. ROC analysis revealed a TAD cutoff value of 31.3 mm (Table 7), as shown in Figure 8.

**Table 6:** Results of multivariate logistic regression analysis model affecting cutout.

Variables	Regression coefficient	SE	*P*	OR	95% CI
Baumgartner’s criteria	3.496	1.237	**.015**	32.99	2.920–372.84
Cleveland index	−0.800	1.686	.635	0.449	0.016–12.238
Parker’s ratio	0.107	0.065	.103	1.113	0.979–1.265
TAD	0.640	0.473	.176	1.896	0.751–4.789
Constant	−10.396	4.253	.015	0.000	

*P*-values < .05 are shown in bold.

CI = confidence interval, OR = odds ratio, SE = standard error, TAD = tip-apex distance.

**Table 7:** The determination of the threshold value of TAD through ROC analysis.

Risk factor	AUC (95% CI)	*P*	Cutout	Sensitivity (%)	Specificity (%)
TAD	0.829 (0.639–1.000)	**.007**	31.3	83.3	85.1

*P*-values < .05 are shown in bold.

AUC = area under the curve, CI = confidence interval, ROC = receiver operating characteristic, TAD = tip-apex distance.

## 4. Discussion

The incidence of ITFs is increasing because of the growing elderly population.^[5]^ Stabilizing unstable ITFs with proper fixation is crucial for restoring patients to their pre-fracture activity levels and reducing the risk of reoperation, complications, and mortality.^[12,13]^

Many nonsurgical factors such as age, body mass index (BMI), Charlson comorbidity index, cognitive status, and time to surgery have been associated with functional outcomes after PFN fixation.^[14]^ In our study, although radiological parameters such as TAD, Cleveland index, and reduction quality were comparable between the groups, patients treated with IPFNA demonstrated significantly higher HHSs. This may be explained by the implant’s claw-shaped antirotational design and a slightly larger collodiaphyseal angle, which may provide improved stability in osteoporotic bone. Additionally, shorter operative times in the IPFNA group may have facilitated faster rehabilitation. Together, these factors may have accounted for the superior functional outcomes observed in this group.

Various types of intramedullary nails are commonly used to treat these fractures.^[9,12,13,15]^ Intramedullary implants have demonstrated better outcomes in unstable fractures, contributing to a growing interest in these devices.^[16]^ However, complications such as cutout, pull-out, varus collapse, and rotational instability have driven the search for improved nail designs.^[20–22]^ In a systematic meta-analysis, Proximal Femoral Nail Antirotation (PFNA; DePuy Synthes), Gamma 3 (Stryker Trauma GmbH), and InterTAN (Smith & Nephew, Inc.) nails were compared, and it was reported that there was no difference between their functional and general complications, but the InterTAN nail could be preferred due to lower revision rates.^[23]^ In another systematic meta-analysis, proximal femoral bionic nails and PFNA were evaluated, and no difference was found between them in terms of complications.^[24]^ A study comparing PFNA and PFN indicated that PFNA is more rotationally stable and has better functional outcomes.^[25]^ Another study compared InterTAN with PFNA, stating that there was no significant superiority and that the decision should be made based on the patient’s general condition.^[26]^

In our study, A-PFN and IPFNA differed in their proximal geometry, lag screw design, and compressive mechanisms. The A-PFN provides controlled compression through a sliding lag screw and a 125° neck-shaft angle, whereas the IPFNA utilizes a claw-shaped interlocking design with a 130° angle, potentially offering greater initial rotational stability. These variations may account for the operative and functional differences observed between the 2 groups, even in the absence of significant radiographic disparities.

Cutout is a significant complication of PFN. Cutout rates ranging from 1.5% to 14.9% have been reported in the literature.^[20,27]^ Many studies have identified BMI, quality of reduction, gender, osteoporosis, screw position, and TAD as risk factors for cutout.^[28,29]^ Additionally, research suggests that women may have a higher risk of cutout due to differences in bone mineral density and strength compared to men.^[30,31]^ Our study’s cutout rate of 5.6% aligns with existing literature, indicating the reliability of our findings. Univariate logistic regression analysis revealed that Baumgartner’s criteria, PR, the Cleveland index, and TAD can significantly affect the risk of cutout.

Many studies have suggested a potential link between osteoporosis and mechanical issues in proximal femoral nailing.^[32,33]^ However, our study did not yield significant results in the risk analysis involving gender and osteoporosis. Proper lag screw positioning is crucial for preventing femoral head perforations.^[34–36]^ The evaluation of nail positioning within the bone involves metrics such as TAD, PR, and Cleveland quadrants.^[37,38]^ A PR exceeding 66% indicates inadequate fixation, while a ratio below 33% indicates stable fixation.^[38]^ Our study identified PR as a key factor in cutout risk reduction when positioned optimally.

Another important parameter in implant cutout assessment is the development of a Cleveland quadrant. Proper positioning based on Cleveland quadrants has been shown to reduce the risk of screw cutting through the femoral head.^[21,29,35]^ Our study found that screws not in the ideal position were significantly associated with cutout risk. Therefore, emphasis should be placed on central-inferior positioning on anteroposterior radiographs and central-posterior positioning on lateral radiographs.

In many studies, a TAD of >25 mm has been identified as a significant risk factor for cutout.^[31,36,38–40]^ However, there are conflicting findings regarding the specific TAD values that serve as cutoff points.^[34,35]^ Another study suggests that increased TAD values may not directly contribute to implant loosening. Coviello et al^[41]^ established a cutoff value of 29.50 mm for TAD and demonstrated that successful fracture reduction did not increase the risk of implant loosening even with higher TAD values.

Although previous studies have identified 25 mm as the critical TAD threshold, our ROC-derived value of 31.3 mm suggests that in elderly osteoporotic patients treated with A-PFN or IPFNA, the cutout risk may become clinically significant at a slightly higher margin. This finding reinforces the well-established role of TAD as a predictor of implant failure, while indicating that in our elderly osteoporotic cohort, the critical threshold may be slightly higher than the classic 25 mm. However, while the 25-mm rule remains a valuable guideline, our findings demonstrate that implant design, patient characteristics, and fracture stability may influence the precise cutoff value. Therefore, our findings extend rather than refute the existing literature and highlight the need for further prospective validation.

Sharma et al^[42]^ emphasized that poor positioning and inadequate reduction of the lag screw cannot be compensated for by any implant. However, implants with antirotation helical blades exhibit superior performance, particularly in the case of osteoporosis.^[43]^ Gunay et al^[27]^ conducted a study comparing the Dyna Locking Trochanteric nail (DLT; U&I Corporation), which features a claw mechanism similar to that of IPFNA, in patients over 80 years of age with stable and unstable ITFs. They reported a 14.9% cutout rate in unstable fractures, indicating that the claw system may not function optimally in such cases, with reduction quality being the most important factor. In another study, Temiz et al^[44]^ assessed DLT nails in 31 elderly patients and found no cutout cases despite varus collapse in 3 patients. In addition, Kim et al^[45]^ compared 3 types of lag screws and found no significant differences in complications, such as cutout, underscoring the importance of fracture reduction and surgical technique.

In the current study, although both A-PFN and IPFNA implants demonstrated comparable radiological outcomes in terms of fracture reduction, TAD, and implant positioning, the IPFNA group exhibited significantly higher HHS and shorter operative times. These findings suggest that while radiological efficacy is similar, the IPFNA implant may offer better short-term functional outcomes and intraoperative convenience.

## 5. Conclusion

In this comparative study of elderly patients with intertrochanteric femoral fractures, both the A-PFN and IPFNA implants provided effective and radiologically comparable fixation. The most clinically relevant message of our findings is that reduction quality, rather than implant design, was the decisive factor: it was the only independent determinant of cutout on multivariate analysis, outweighing implant type. Beyond this, the IPFNA group achieved a significantly higher HHS (85.5 vs 77.5, *P* < .001) together with a shorter operative time, which suggests a short-term functional and intraoperative advantage for this implant. With respect to fixation safety, ROC analysis identified a TAD threshold of 31.3 mm – slightly higher than the classic 25-mm rule – above which the cutout risk increased markedly in this elderly osteoporotic cohort, while optimal screw positioning according to the Cleveland index and an appropriate PR further contributed to reducing failure risk. Taken together, these findings emphasize that meticulous anatomical reduction and accurate screw placement remain the principal determinants of a successful outcome, whereas the choice between these 2 antirotational implants appears to play a secondary role. Given the retrospective design and the small number of cutout events, these results – and particularly the 31.3-mm TAD threshold – should be regarded as hypothesis-generating and warrant validation in larger prospective, multicenter studies.

## 6. Limitations

This study had several limitations. First, it had a retrospective design and lacked evaluations of BMI and pre-fracture mobility scores, with a small sample size, potentially impacting reliability. Second, the absence of bone mineral density assessment through DXA scanning may have affected the precision of osteoporosis diagnosis. Instead, radiographic criteria were used, which, although validated, may not reflect actual bone mineral density. The lack of these parameters may partially explain why the ROC-derived TAD cutoff value (31.3 mm) in our study differed from the value reported in the literature. Third, although the 2 treatment groups differed in sample size, an a priori power analysis confirmed that the overall sample size was sufficient to detect significant differences. Nonetheless, an unequal distribution could introduce minor statistical bias, which is a limitation of this study. Fourth, there was no pre-injury HHS or documented baseline mobility levels. This omission, owing to retrospective data limitations, may affect the interpretation of the postoperative functional outcomes. Fifth, with only 6 cutout events, the logistic regression analyses are exploratory; the resulting odds ratios carry wide confidence intervals and limited statistical power and should be regarded as hypothesis-generating pending validation in larger cohorts. Another limitation is that although the surgeries were performed by experienced surgeons using the same institutional protocol, the involvement of multiple surgeons may still lead to minor differences in surgical practice.

## Author contributions

**Conceptualization:** Ömer Can Ünlü, İbrahim Altun, Kürşat Tuğrul Okur.

**Data curation:** Ömer Can Ünlü, İbrahim Altun, Muhammed Melez, Firat Ozan.

**Formal analysis:** Ömer Can Ünlü, İbrahim Altun, Muhammed Melez, Kürşat Tuğrul Okur, Firat Ozan.

**Investigation:** Ömer Can Ünlü, İbrahim Altun, Muhammed Melez.

**Methodology:** Ömer Can Ünlü, İbrahim Altun.

**Software:** İbrahim Altun, Firat Ozan.

**Supervision:** İbrahim Altun, Firat Ozan.

**Visualization:** Muhammed Melez, Kürşat Tuğrul Okur.

**Validation:** Ömer Can Ünlü, Muhammed Melez, Kürşat Tuğrul Okur.

**Writing – original draft:** Ömer Can Ünlü, İbrahim Altun.

**Writing – review & editing:** Ömer Can Ünlü, İbrahim Altun, Firat Ozan.
